# Association between the dietary inflammatory index and markers of endothelial and systemic inflammation in hemodialysis patients

**DOI:** 10.3389/fnut.2023.1230747

**Published:** 2023-09-14

**Authors:** Arman Arab, Elham Karimi, Maryam Nazari, Hadi Tabibi, Atefeh As’habi

**Affiliations:** ^1^Department of Community Nutrition, School of Nutrition and Food Sciences, Isfahan University of Medical Sciences, Isfahan, Iran; ^2^Department of Clinical Nutrition, School of Nutrition and Food Sciences, Isfahan University of Medical Sciences, Isfahan, Iran; ^3^Research Development Center, Arash Women’s Hospital, Tehran University of Medical Sciences, Tehran, Iran; ^4^Food Safety Research Center (Salt), Semnan University of Medical Sciences, Semnan, Iran; ^5^Department of Clinical Nutrition and Dietetics, Faculty of Nutrition and Food Technology, National Nutrition and Food Technology Research Institute, Shahid Beheshti University of Medical Sciences, Tehran, Iran

**Keywords:** inflammation, diet, hemodialysis, Iran, dietary inflammatory index

## Abstract

**Objectives:**

The current survey aimed to investigate the link between energy-adjusted dietary inflammatory index (E-DII) and risk factors for CVD including markers of endothelial and systemic inflammation in Iranian hemodialysis patients.

**Methods:**

Patients on hemodialysis for at least 6 months prior to enrollment were considered eligible in this cross-sectional study. The usual dietary intakes of the hemodialysis individuals were examined through 4 non-consecutive days including 2 dialysis days and 2 non-dialysis days using a 24-h recall approach to calculate E-DII. Multiple linear regression analysis was utilized to investigate the link between E-DII and selected biomarkers of inflammation and oxidative stress including high-sensitive C reactive protein (hs-CRP), serum intercellular adhesion molecule (sICAM), serum vascular cell adhesion molecule (sVCAM), malondialdehyde, and nitric oxide (NO), sE-selectin, and endothelin-1, and beta (β) and 95% confidence interval (CI) was reported. Value of *p* < 0.05 was considered statistically significant.

**Results:**

Overall, 291 hemodialysis patients make up our study population. In the crude model, the E-DII score was positively associated with a higher sVCAM-1 (β = 177.39; 95% CI: 60.51, 294.26; *p*_trend_ = 0.003). Further adjustment for potential confounders attenuated the findings in a way that an increase of 128.72 in the sVCAM-1 was observed when the E-DII score increased from −2.68 to −1.14 (95% CI: 13.50, 243.94). After controlling for potential confounders, E-DII was associated with sE-selectin in hemodialysis patients in the highest category of E-DII as compared to the lowest category (β = 4.11; 95% CI: 0.22, 8.00; *p*_trend_ = 0.039).

**Conclusion:**

The present findings suggest that adherence to a pro-inflammatory diet among hemodialysis patients is associated with a higher inflammatory status as evidenced by sVCAM-1 and sE-selectin; however, bidirectionality may exist and the role of residual confounders should be taken into account. Therefore, more longitudinal investigations are needed to elucidate the role of diet on the inflammatory status of hemodialysis patients.

## Introduction

The prevalence of cardiovascular disease (CVD) has been estimated to be 20 times higher among hemodialysis (HD) patients compared to the general population and also CVD accounts for nearly 50% of deaths in these patients (1–3). Chronic systemic inflammation is known as one of the main mediators of increased CVD risk in HD patients (4–6). Chronic inflammation increases the production of pro-inflammatory cytokines subsequently leading to the synthesis of the markers of systemic inflammation [i.e., C-reactive protein (CRP)] and vascular inflammation [i.e., soluble vascular cell adhesion molecule (sVCAM-1), soluble intercellular adhesion molecule (sICAM-1), and sE-selectin] (7).

Iran, like other developing countries, has experienced a rapid nutritional transition with a great interest in Western-style diet (8). However, undergoing HD may change the usual dietary intakes of individuals with a greater recommendation for high-quality protein and the possible necessity to limit foods and beverages that have lots of sodium, phosphorous, and potassium (9). Diet has an important role in immunonutrition through various nutrients which are suggested to influence inflammatory, immunological, and nutritional parameters (10). The dietary inflammatory index (DII) is a population-based, literature-based dietary scoring tool that provides the potential inflammatory properties of a diet (11). It represents the overall pro−/anti-inflammatory properties of the diet rather than individual foods and nutrients (11). It was shown that a pro-inflammatory diet, as indicated by a higher DII score, is associated with a higher prevalence of chronic kidney disease (CKD) and reduced kidney function (12, 13). The dietary inflammatory index was also shown to be positively associated with higher rates of mortality in HD patients (14). Also, a diet with higher pro-inflammatory potential was suggested to be linked with lower quality of life in patients undergoing HD (15). Moreover, the DII score was shown to be associated with CRP among HD patients (16).

Previous studies suggested an association between DII and CVD risk factors among different study populations (17–20); however, this issue was overlooked in HD patients. Due to the presence of reverse epidemiology phenomena or risk factor reversal, the traditional risk factors of CVD cannot explain the high prevalence of this phenomenon in HD patients (21, 22). Hence, novel risk factors have been suggested by previous studies to be measured in these patients for determining the risk of CVD (4, 5).

This study seeks to investigate the inflammatory potential of diet, calculated by DII, concerning risk factors for CVD including markers of endothelial and systemic inflammation. The findings of this study can be used to inform health agencies regarding the possible role of diet in reducing the CVD risk factors of HD patients.

## Methods

### Population and study design

In the current cross-sectional study, adult HD patients from 50 HD centers in Tehran, Iran, were evaluated sequentially from August 2019 to June 2020. First, the list of all the HD centers in Tehran was obtained from the Iran Dialysis Center, and then by referring to each of the 50 HD centers in Tehran, the names of all the HD patients were taken, and then the names of the patients who met the eligibility criteria to be enrolled in this study were recorded (*n* = 2,302). Second, the names of HD centers in Tehran were sorted alphabetically, and then the names of the patients in these centers were listed. Finally, 291 out of 2,302 subjects were selected using the systematic sampling method. Adult subjects (age ≥ 18 years) on HD for at least 6 months prior to enrollment were included. A history of HIV infection, malignancies, chronic or acute pancreatitis, liver disease, and inflammatory diseases were considered as the exclusion criteria. All patients were on HD three times a week (4 h per session) via bicarbonate dialysate and polysulfone capillary dialyzers. All of the enrolled HD patients provided written informed consent forms. Upon approval by the Ethics Committee of the National Nutrition and Food Technology Research Institute of Iran (IR.SBMU.NNFTRI.REC.1387.319), the study was initiated under the Declaration of Helsinki.

### Dietary assessment

The usual dietary intakes of the HD individuals were examined through 4 non-consecutive days including 2 dialysis days and 2 non-dialysis days using a 24-h recall approach. Since dietary intakes of patients may be different on dialysis vs. non-dialysis days, both days were selected to capture day-to-day variation in diet (23). Through a face-to-face interview with a trained dietitian, participants were asked to recall all the drinks and food items consumed within 24 h. Portion sizes models were used to help people in estimating portion size and improve accuracy. Using Nutritionist IV software (First Databank® Inc., Hearst Corp., San Bruno, CA, United States) and the USDA food and nutrient database (24), dietary intakes were analyzed to determine the daily intakes of energy, macronutrients, and micronutrients of HD patients.

### DII calculation

We used an approach suggested by Shivappa et al. to calculate energy-adjusted DII (E-DII). Before the E-DII calculation, the energy-adjusted amount of each food item was calculated using the residual technique (25). Of 45 dietary items suggested by Shivappa et al., 28 food items were available for E-DII calculation including vitamins A, D, E, B1, B2, B3, B6, B9, B12, C, β-carotene, n-3 fatty acids, n-6 fatty acids, cholesterol, saturated fatty acids (SFA), trans fatty acids (TFA), polyunsaturated fatty acids (PUFA), mono-unsaturated fatty acids (MUFA), magnesium, zinc, iron, selenium, caffeine, dietary fiber, carbohydrate, fat, protein, and energy (11). Initially, subjects’ dietary consumption was subtracted from the “standard global mean” and then it was divided by the “global standard deviation” to calculate the Z score of each dietary parameter. Subsequently, the Z score of each food item was transformed to the centered percentile to minimize data skewness and then multiplied by the score of inflammatory properties of each food item. Lastly, the overall E-DII for each participant was calculated by summing up the inflammatory scores of 28 food items calculated previously. Shivappa et al. suggested a DII score range of −8.87 to +7.98 with higher DII values representing a diet with pro-inflammatory properties, while lower values indicate a diet with anti-inflammatory features (11).

### Biochemical parameters

A venous blood sample (10 mL) was obtained from each patient before dialysis and after 12–14 h of fasting. Then, blood samples were centrifuged at 2000 rpm for 10 min to separate serum and subsequently, the extracted serum was transferred to sterile microtubes and stored at −70°C until the time of biochemical analysis. Serum albumin, urea, and creatinine were assessed using Selectra 2 Autoanalyzer (Vital Scientific, Spankeren, the Netherlands) employing commercial kits (Pars-Azmoon, Tehran, Iran) with the intra- and inter-assay coefficients of variation (CV) < 3%. The concentration of serum endothelin-1 was examined via ELISA kits (Biomedica, Vienna, Austria), with an intra- and inter-assay CV of 8.5%. Serum concentrations of malondialdehyde (MDA) and nitric oxide (NO) were measured using a colorimetric approach via commercial kits (Cayman Chemical, Ann Arbor, MI, United States), with the intra- and inter-assay CV of 4.6% and 7.8%, respectively. The serum concentrations of sE-selectin, sVCAM-1, and sICAM-1 were measured via ELISA kits (Diaclone, Besancon, France) with the intra- and inter-assay CV of 6.7, 6.3, and 3.5%, respectively. The serum concentration of hs-CRP was determined using ELISA kits (Diagnostics Biochem Canada, London, Canada) with an intra- and inter-assay CV of 4.6%.

### Assessment of confounders

The body mass index (BMI) was calculated using participants’ weight and height, measured at the end of their dialysis session. Dialysis vintage was outlined as the time that each patient was on HD and stated as a year. Dialysis adequacy was calculated, based on the Kt/V index, using dialysis length, post-dialysis weight, ultrafiltration volume, and pre-and post-dialysis serum urea concentration (26).

### Statistical analysis

Using a suggested formula for sample size calculation of cross-sectional studies from small populations and with *α* = 0.05 and *d* = 0.05, a total sample of 292 was calculated (27). The statistical analysis was done using SPSS version 26 (IBM Corp., Armonk, NY, United States). The normality distribution of continuous variables was assessed using the skewness statistic, Q-Q plot, and Kolmogorov–Smirnov test. Continuous and categorical variables are presented as mean ± standard error (SE) and number (percent), respectively. The differences in quantitative variables across tertiles of E-DII were assessed via the one-way analysis of variance (ANOVA). The distribution of qualitative variables across tertiles of E-DII was examined using the Chi-squared test. A residual approach was implemented to calculate energy-adjusted values of food items. Multiple linear regression analysis was utilized to investigate the link between E-DII and selected biomarkers of inflammation and oxidative stress in three different models. In the first model, gender, and age (continuous) were entered. In the second model, further adjustment was made for albumin (continuous), serum urea (continuous), serum creatinine (continuous), dialysis vintage (continuous), and dialysis adequacy (continuous). BMI (continuous) was adjusted in the final model. Value of *p* < 0.05 was considered statistically significant and all analyses were done two-tailed.

## Results

A total of 291 HD patients (127 females and 164 males) with a mean (SE) age of 57.73 (0.88) years, BMI of 24.11 (0.26) kg/m^2^, dialysis adequacy of 1.23 (0.02) Kt/V, and dialysis vintage of 4.27 (0.25) years make up our study population. Of 291 HD patients, 261 participants had arteriovenous (AV) fistula and others had a central venous catheter. Participants of the highest tertile of E-DII compared to the lowest tertile significantly had higher sVCAM-1 and were more likely to be younger (*p* < 0.05). No significant differences were observed in terms of other baseline variables across tertiles of E-DII score (*p* > 0.05; Table 1).

**Table 1:** Characteristics of the study population stratified by tertiles of dietary inflammatory index.

Variables	Tertiles of dietary inflammatory index			
	T1 [<−2.25]	T2 [−2.25 to − 1.56]	T3 [> − 1.56]	*p*-value
*N*	97	97	97	
DII	−2.68 ± 0.03	−1.87 ± 0.01	−1.14 ± 0.03	<0.001
*General information*				
Age (year)	60.48 ± 1.44	57.96 ± 1.66	54.74 ± 1.47	0.030
Female	46 (47.4)	48 (49.5)	33 (34.0)	0.060
Weight (kg)	63.73 ± 1.34	64.06 ± 1.36	64.31 ± 1.45	0.956
Height (m)	1.61 ± 0.01	1.63 ± 0.009	1.63 ± 0.01	0.234
BMI (kg/m^2^)	24.40 ± 0.43	24.06 ± 0.46	23.86 ± 0.45	0.697
Dialysis adequacy (Kt/V)	1.25 ± 0.03	1.24 ± 0.04	1.20 ± 0.04	0.630
Dialysis vintage (year)	3.75 ± 0.45	4.14 ± 0.43	4.91 ± 0.46	0.183
Serum creatinine (mg/dL)	8.35 ± 0.29	8.98 ± 0.35	9.09 ± 0.33	0.230
Serum urea (mg/dL)	120.32 ± 3.25	122.10 ± 3.11	126.21 ± 2.50	0.357
Albumin (g/dL)	4.14 ± 0.03	4.11 ± 0.04	4.10 ± 0.03	0.758
*Inflammatory biomarkers*				
hs-CRP (mg/L)	5.62 ± 0.32	5.37 ± 0.32	5.65 ± 0.31	0.807
sICAM-1 (ng/mL)	645.06 ± 26.95	649.21 ± 27.19	669.15 ± 21.93	0.774
sVCAM-1 (ng/mL)	728.35 ± 40.14	737.64 ± 39.12	905.74 ± 45.73	0.004
sE-selectin (ng/mL)	23.14 ± 1.38	25.20 ± 1.49	25.68 ± 1.48	0.420
MDA (μmol/L)	5.92 ± 0.13	5.63 ± 0.08	5.85 ± 0.12	0.190
NO (μmol/L)	43.03 ± 1.12	42.49 ± 0.94	42.05 ± 1.01	0.797
Endothelin-1 (pg/mL)	25.10 ± 2.46	31.10 ± 2.87	26.71 ± 2.69	0.264

Data are presented as mean ± standard error or number (% within tertiles of the dietary inflammatory index).

*p* value obtained from ANOVA for continuous variables and Chi-squared test for categorical variables.

BMI, Body mass index; hs-CRP, High-sensitive C reactive protein; sICAM, Serum intercellular adhesion molecule; sVCAM, Serum vascular cell adhesion molecule; MDA, Malondialdehyde; NO, Nitric oxide; LP, Lipoprotein; TG, Triglyceride; TC, Total cholesterol; HDL, High-density lipoprotein; LDL, Low-density lipoprotein; and DII, Dietary inflammatory index.

Dietary intakes of HD patients according to tertiles of E-DII are presented in Table 2. As can be seen, participants in the greatest tertile of E-DII consumed higher amounts of total energy and SFA, as well as lower amounts of protein, total fiber, PUFA, magnesium, selenium, vitamin E, A, C, B1, B2, B3, B6, and folic acid (*p* < 0.05).

**Table 2:** Selected nutrients intake of participants across tertiles of dietary inflammatory index.^*^

Variables	Tertiles of dietary inflammatory index			
	T1	T2	T3	*p*-value
Total energy (kcal/day)^**^	1168.60 ± 45.17	1276.17 ± 52.63	1524.74 ± 47.11	<0.001
Protein (g/day)	51.17 ± 0.94	51.44 ± 1.14	47.27 ± 0.92	0.005
Fat (g/day)	37.08 ± 0.94	37.71 ± 1.03	36.93 ± 1.13	0.854
Carbohydrate (g/day)	194.96 ± 3.21	195.16 ± 2.48	201.16 ± 2.67	0.210
Total fiber (g/day)	9.37 ± 0.55	6.88 ± 0.18	6.07 ± 0.17	<0.001
SFA (g/day)	9.02 ± 0.29	9.05 ± 0.28	10.38 ± 0.47	0.012
PUFA (g/day)	12.99 ± 0.52	12.93 ± 0.55	11.19 ± 0.51	0.027
MUFA (g/day)	10.41 ± 0.34	10.85 ± 0.40	10.87 ± 0.45	0.665
Magnesium (mg/day)	123.87 ± 2.77	106.57 ± 1.93	88.65 ± 2.39	<0.001
Zinc (mg/day)	4.71 ± 0.11	4.46 ± 0.12	4.27 ± 0.16	0.080
Iron (mg/day)	10.10 ± 0.27	9.43 ± 0.18	9.78 ± 0.32	0.203
Selenium (μg/day)	1.16 ± 0.28	0.69 ± 0.02	0.53 ± 0.01	0.026
Vitamin E (mg/day)	2.86 ± 0.24	2.00 ± 0.18	1.16 ± 0.10	<0.001
Vitamin A (RE/day)	447.31 ± 47.82	270.66 ± 21.36	227.98 ± 28.12	<0.001
Vitamin C (mg/day)	51.45 ± 2.91	31.93 ± 2.09	20.13 ± 1.87	<0.001
Vitamin B1 (mg/day)	1.35 ± 0.01	1.33 ± 0.01	1.29 ± 0.01	0.048
Vitamin B2 (mg/day)	0.86 ± 0.02	0.79 ± 0.01	0.78 ± 0.02	0.028
Vitamin B3 (mg/day)	16.93 ± 0.33	17.05 ± 0.39	15.59 ± 0.34	0.007
Vitamin B6 (mg/day)	1.19 ± 0.26	0.69 ± 0.02	0.52 ± 0.02	0.006
Vitamin B12 (μg/day)	4.57 ± 1.85	1.74 ± 0.13	2.01 ± 0.30	0.130
Folic acid (μg/day)	121.30 ± 6.08	92.72 ± 3.01	80.11 ± 3.24	<0.001

Data are presented as mean ± standard error and obtained from ANOVA.

*p* < 0.05 was considered statistically significant. ^*^All values have been adjusted for total energy intake using a residual method. ^**^Energy intake was not adjusted. SFA, Saturated fatty acids; PUFA, Polyunsaturated fatty acids; MUFA, Monounsaturated fatty acids; RE, Retinol equivalents.

The relation between E-DII and selected biomarkers of inflammation and oxidative stress is represented as beta estimates and the corresponding 95% CIs and can be seen in Table 3. In the crude model, the E-DII score was positively associated with a higher sVCAM-1 (β = 177.39; 95% CI: 60.51, 294.26; *p*_trend_ = 0.003) in individuals in the highest tertile of E-DII as compared to those in the lowest tertile. Adjustments for sex and age attenuated the findings (β = 15.64; 95% CI: 47.01, 284.28; *p*_trend_ = 0.007). Further adjustment for dialysis adequacy, dialysis vintage, serum creatinine, serum urea, and albumin attenuated the findings more, however, E-DII was still significantly associated with sVCAM-1 in participants in the third tertile of E-DII as compared to those in the first tertile (β = 128.63; 95% CI: 13.48, 243.78; *p*_trend_ = 0.029). After controlling for BMI, an increase of 128.72 in the sVCAM-1 was observed when the E-DII score increased from −2.68 to −1.14 (95% CI: 13.50, 243.94; *p*_trend_ = 0.029) There was no significant association between sE-selectin and E-DII in the crude model (β = 2.54; 95% CI: −1.45, 6.53; *p*_trend_ = 0.212) or after adjustment for age and sex (β = 3.53; 95% CI: −0.47, 7.54; *p*_trend_ = 0.085) for those in the highest tertile of E-DII compared to lowest tertile. However, further adjustment for dialysis adequacy, dialysis vintage, serum creatinine, serum urea, albumin, and BMI made this association significant (β = 4.11; 95% CI: 0.22, 8.00; *p*_trend_ = 0.039). No significant relationship was detected between E-DII and hs-CRP, sICAM-1, MDA, NO, and Endothelin-1 (Table 3).

**Table 3:** Beta (β) and 95% confidence interval for biomarkers of inflammation and oxidative stress across tertiles of the dietary inflammatory index.

	Tertiles of dietary inflammatory index			
	T1	T2	T3	*p*-trend
hs-CRP (mg/L)				
Crude	Ref	−0.24 (−1.14, 0.65)	0.02 (−0.86, 0.92)	0.954
Model 1	Ref	−0.14 (−1.02, 0.74)	0.19 (−0.70, 1.08)	0.682
Model 2	Ref	−0.20 (−1.11, 0.70)	0.17 (−0.70, 1.05)	0.701
Model 3	Ref	−0.18 (−1.09, 0.72)	0.16 (−0.72, 1.04)	0.722
sICAM-1 (ng/mL)				
Crude	Ref	4.15 (−66.12, 74.43)	24.09 (−46.00, 94.18)	0.501
Model 1	Ref	−5.05 (−71.54, 61.42)	34.22 (−32.67, 101.11)	0.319
Model 2	Ref	−7.08 (−77.02, 62.86)	24.52 (−43.25, 92.31)	0.479
Model 3	Ref	−9.32 (−79.03, 60.38)	26.26 (−41.26, 93.80)	0.448
sVCAM-1 (ng/mL)				
Crude	Ref	9.29 (−107.88, 126.47)	177.39 (60.51, 294.26)	0.003
Model 1	Ref	4.51 (−112.71, 121.74)	165.64 (47.01, 284.28)	0.007
Model 2	Ref	49.25 (−69.17, 167.69)	128.63 (13.48, 243.78)	0.029
Model 3	Ref	49.14 (−69.42, 167.70)	128.72 (13.50, 243.94)	0.029
sE-selectin (ng/mL)				
Crude	Ref	2.05(−1.94, 6.05)	2.54 (−1.45, 6.53)	0.212
Model 1	Ref	2.66 (−1.33, 6.66)	3.53 (−0.47, 7.54)	0.085
Model 2	Ref	3.03 (−0.99, 7.05)	4.00 (0.10, 7.90)	0.044
Model 3	Ref	3.21 (−0.81, 7.24)	4.11 (0.22, 8.00)	0.039
MDA (μmol/L)				
Crude	Ref	−0.20 (−0.45, 0.05)	−0.06 (−0.32, 0.19)	0.624
Model 1	Ref	−0.18 (−0.44, 0.06)	0.008 (−0.25, 0.26)	0.970
Model 2	Ref	−0.16 (−0.44, 0.11)	0.06 (−0.21, 0.33)	0.661
Model 3	Ref	−0.16 (−0.44, 0.11)	0.06 (−0.21, 0.33)	0.676
NO (μmol/L)				
Crude	Ref	−0.54 (−3.38, 2.30)	−0.98 (−3.82, 1.86)	0.499
Model 1	Ref	−0.83 (−3.66, 1.98)	−1.59 (−4.45, 1.26)	0.274
Model 2	Ref	−0.28 (−3.32, 2.76)	−1.14 (−4.11, 1.81)	0.447
Model 3	Ref	−0.11 (−3.13, 2.91)	−1.24 (−4.18, 1.69)	0.407
Endothelin-1 (pg/mL)				
Crude	Ref	5.99 (−1.40, 13.39)	1.60 (−5.77, 8.98)	0.671
Model 1	Ref	5.51 (−1.86, 12.90)	1.15 (−6.31, 8.62)	0.750
Model 2	Ref	5.26 (−2.75, 13.27)	0.91 (−6.87, 8.70)	0.817
Model 3	Ref	5.28 (−2.73, 13.30)	0.89 (−6.90, 8.68)	0.818

Data are presented as β (95% confidence interval) and obtained from linear regression. *p* < 0.05 was considered statistically significant. Crude: Unadjusted. Model 1: Adjusted for age and sex. Model 2: Model 1+ dialysis adequacy, dialysis vintage, serum creatinine, serum urea, and albumin. Model 3: Model 2 + body mass index. hs-CRP, High-sensitive C reactive protein; sICAM, Serum intercellular adhesion molecule; sVCAM, Serum vascular cell adhesion molecule; MDA, Malondialdehyde; NO, Nitric oxide.

## Discussion

A population of Iranian HD patients was recruited to examine the association between E-DII and markers of endothelial and systemic inflammation as novel risk factors for CVD. We found that E-DII can be a predictor of higher sVCAM-1 and sE-selectin. Our findings suggest the importance of the inflammatory potential of diet in the context of endothelial and systemic inflammation. New and substantial information regarding the association of E-DII with novel CVD risk factors in a sample of Iranian HD patients can be added to the literature by these findings.

In the current study, E-DII and sVCAM-1 were associated independently of age and sex. However, the inclusion of other confounders (i.e., dialysis adequacy, dialysis vintage, serum creatinine, serum urea, albumin, and BMI) attenuated the findings, indicating that the potential effect of E-DII may be related to body composition, residual kidney function, and other aspects of dialysis, as evidenced by previous literature (28–30). Moreover, patients in the highest tertile of E-DII tended to have higher sE-selectin that was independent of assessed confounders. The findings regarding sVCAM-1 and sE-selectin also imply that a larger sample size may amplify the observed association. Therefore, further studies with a larger sample size are needed to elucidate this issue.

According to the available literature, no research has been carried out to investigate the association between E-DII and markers of endothelial and systemic inflammation as novel risk factors for CVD among Iranian HD patients. A cross-sectional study on 221 patients (mean age of 56.57 years) diagnosed with CKD among the Iranian population proposed that compliance with a pro-inflammatory diet was associated with a higher risk of CKD progression (odds ratio: 2.12; 95%CI: 1.05, 4.26) (13). Another population-based study among 21,649 subjects (47.3 years) also concluded that a diet with higher inflammatory potentials is associated with higher CKD prevalence and declining kidney function (12). Another cross-sectional study was done on 105 HD patients (57.5 years) at Turkish dialysis centers and suggested that DII was significantly correlated with the markers of malnutrition and inflammation (e.g., CRP) (16). A cohort study of 137 patients undergoing HD (61.7 years) revealed a direct association between DII and mortality during the 2-year follow-up (14). Another study among 532 adolescents (12–17 years old) in European countries also suggested a positive association between DII and sVCAM-1 (31). There are some points worthy of mention. There is no previous study that can be used to confirm the present findings in HD patients. Moreover, the available literature is heterogeneous in terms of the study population, age, ethnicity, background comorbidities, sample size, DII calculation, and methodological settings that make the comparison difficult. Furthermore, previous studies used various approaches to assess patients’ dietary intakes (e.g., food frequency questionnaire, food record, and food recall) which adds to the existing heterogeneity. Overall, this study, similar to other available evidence, confirms the possible link between diet and inflammatory status (32).

As evidenced by a majority of previous documents, CKD is accompanied by a low-grade systemic inflammation that increases the secretion of inflammatory cytokines which led to an inflammatory state (33). The etiology of inflammation among HD patients is multifactorial and complex, including increased oxidative stress, malnutrition, subclinical or clinical infection of the vascular access port, decreased clearance and increased release of inflammatory cytokines, oxidative and carbonyl stress, frequent contact between blood mononuclear cells and dialysis tubes and dialyzer membranes, and impure dialysis water/solution (34). However, as evidenced by the findings of the current study, a pro-inflammatory diet, as shown by higher E-DII values, might be another reason for inflammation. The findings of the current study reinforce the idea that a diet poor in anti-inflammatory parameters, and rich in pro-inflammatory parameters may increase inflammation among HD patients. As a new approach to represent the inflammatory potential of the diet, DII can be implemented in a diverse population with available dietary data (11, 35). The DII can also be used to assess individuals’ dietary inflammatory status and subsequently guide them in reducing inflammation levels to decrease the risk of certain chronic conditions (11). Dietary components with anti-oxidative and anti-inflammatory properties are proposed to be effective in reducing inflammation and CVD risk (36). Potential agents with anti-oxidative and anti-inflammatory properties that have been studied in CKD patients to decrease the levels of inflammation are fish oil, vitamin A/carotenoids, vitamin C, and vitamin E (37). Similarly, in the current study, dietary vitamin E (*p* < 0.001), vitamin C (*p* < 0.001), and vitamin A (*p* < 0.001) intakes were higher in the first tertile (more anti-inflammatory diet). Moreover, it has been reported that high glycemic index foods and SFA are associated with elevated levels of inflammation (38). In line with this, patients with the highest E-DII consumed higher amounts of SFA (*p* = 0.012) and lower amounts of PUFA (*p* = 0.027).

The total energy intakes of our study population ranged from 1168.60 to 1524.74 kcal/day across tertiles of E-DII. This finding implies that the included participants consumed lower amounts of energy as indicated by their BMI status which was 18.33–23.70 kcal/kg/day. A previous study by Bossola et al. also proposed that 70.2% of their HD patients had inadequate energy intake (39). A dietary energy intake of 30–35 kcal/kg/day is generally recommended for HD patients; however, various reports revealed that the actual energy intake of HD patients is lower than recommended values (40–42). Psychosocial modifiers, low diet quality, poor appetite, and low dialysis adequacy are among the most suggested factors contributing to lower dietary intake and malnutrition among HD patients (42). In agreement with improper total energy intake, dietary consumption of other nutrients and food groups also failed to reach recommended values contributing to the lower diet quality and possibly higher inflammatory potential of a diet (43). Previous reports suggested a higher intake of fruits, vegetables, whole grains, nuts, seeds, legumes, and spices as well as a lower intake of red meat, processed meat, refined grains, added sugars, and fried foods. Moreover, healthy fats should be chosen including olive oil, avocado, fish oil, and flaxseed oil (44). However, all of these recommendations are not practical for those undergoing HD, and a personalized dietary recommendation is strongly suggested for this population.

### Strengths and limitations

This is the first study among Iranian HD patients that examined the association between E-DII and comprehensive markers of endothelial and systemic inflammation as novel risk factors for CVD, which can be the main strength of the current survey.

The present study has several limitations. We used a 4-day dietary recall to obtain the dietary intakes of participants, however, this approach mainly relies on subjects’ memory and therefore might be prone to over−/underestimation of usual dietary intakes. Therefore, it is strongly suggested that future studies use multiple pass 24-h recall to overcome some limitations of the classical 24-h approach. A total of 28 out of 45 dietary items were available for calculating E-DII in which herbs and spices were overlooked including ginger, saffron, turmeric, pepper, rosemary, thyme, and oregano. Likewise, a cause-and-effect conclusion cannot be obtained due to the cross-sectional design of this paper. All patients suffered from CKD; however, the underlying causes were ignored in collecting data and therefore might be another limitation of the current study. Moreover, other co-existing diseases including diabetes, hypertension, thyroid disorders, etc. should also be considered in collecting data in future studies.

## Conclusion

The present findings suggest that adherence to a pro-inflammatory diet among HD patients is associated with a higher inflammatory status as evidenced by sVCAM-1 and sE-selectin; however, bidirectionality may exist and the role of residual confounders should be taken into account. Therefore, more longitudinal investigations are needed to elucidate the role of diet on the inflammatory status of HD patients. Likewise, further studies with a larger sample size are also needed to confirm our findings.

## Data availability statement

The raw data supporting the conclusions of this article will be made available by the authors, without undue reservation.

## Ethics statement

The studies involving humans were approved by The National Nutrition and Food Technology Research Institute of Iran Ethics Committee accepted the study protocol as satisfactory (IR.SBMU.NNFTRI.REC.1387.319). The studies were conducted in accordance with the local legislation and institutional requirements. The participants provided their written informed consent to participate in this study.

## Author contributions

AtA and HT: conception and design and acquisition of data. ArA: analysis and interpretation of data. ArA, EK, AtA, and MN: drafting. ArA, EK, AtA, and HT: intellectual content revision. All authors contributed to the article and approved the submitted version.

## Conflict of interest

The authors declare that the research was conducted in the absence of any commercial or financial relationships that could be construed as a potential conflict of interest.

## Publisher’s note

All claims expressed in this article are solely those of the authors and do not necessarily represent those of their affiliated organizations, or those of the publisher, the editors and the reviewers. Any product that may be evaluated in this article, or claim that may be made by its manufacturer, is not guaranteed or endorsed by the publisher.
